# Supplementary material for: Judging the difficulty of perceptual decisions
Source: bioRxiv. 2023 Jun 5:2023.02.13.528254. Originally published 2023 Feb 13. Preprint. [Version 2] doi: 10.1101/2023.02.13.528254 (PMC9949003; doi:10.1101/2023.02.13.528254)
Supplement: Supplement 1 [file NIHPP2023.02.13.528254v2-supplement-1.pdf]

bioRxiv preprint doi: https://doi.org/10.1101/2023.02.13.528254; this version posted June 5, 2023. The copyright holder for this preprint (which was not certified by peer review) is the author/funder, who has granted bioRxiv a license to display the preprint in perpetuity. It is made available under aCC-BY 4.0 International license.

Supporting Information

.

Subj	κ	u	a	d	C_0	μ_{nd}
1	5.89	2.88	0.17	-2.41	-0.02	0.39
2	4.50	2.73	0.22	-0.15	0.10	0.41
3	4.67	1.57	1.23	1.39	-0.02	0.48
4	4.89	1.03	6.00	3.45	0.02	0.49
5	4.01	0.91	6.00	2.92	-0.05	0.51
6	1.34	2.38	0.74	2.90	0.05	0.40
7	4.92	3.80	0.28	-3.00	-0.04	0.43
8	5.36	2.23	0.39	0.68	0.07	0.41
9	2.97	1.82	-0.16	1.76	0.03	0.51
10	5.20	1.25	0.88	4.21	0.01	0.39
11	6.25	1.26	1.06	3.70	0.05	0.37
12	4.55	4.53	0.94	-0.88	-0.03	0.42
13	5.82	4.92	0.36	-1.65	0.02	0.53
14	4.72	0.82	6.00	3.44	0.03	0.55
15	4.27	1.21	0.96	2.72	-0.02	0.42
16	4.53	1.19	1.39	4.18	-0.02	0.51
17	4.23	4.82	0.50	-3.00	0.06	0.50
18	5.73	3.29	0.36	-3.00	-0.08	0.42
19	5.21	5.00	0.65	-1.35	0.00	0.38
20	4.87	3.14	-0.24	5.00	0.10	0.53
Mean	4.70	2.54	1.39	1.05	0.01	0.45

Table S1. Fit parameter values for drift diffusion model of color judgments (Exp. 1a).

Subj	κ	u	a	d	μ_{nd}
1	3.98	1.22	5.00	1.31	0.25
2	3.65	1.50	4.64	1.54	0.19
3	2.97	0.97	-1.02	-0.49	0.40
4	3.88	1.54	-0.81	-0.13	0.36
5	3.82	1.18	2.45	0.73	0.34
6	1.94	1.46	3.34	1.90	0.38
7	3.20	1.63	1.08	1.11	0.32
8	3.53	1.18	2.47	4.00	0.19
9	2.15	0.72	-1.50	-1.75	0.42
10	2.64	1.29	4.78	1.45	0.27
11	2.40	1.36	4.98	1.94	0.11
12	2.76	1.63	1.56	1.67	0.32
13	2.50	1.39	4.85	1.55	0.21
14	2.92	1.36	1.15	1.93	0.24
15	3.76	1.12	4.79	1.87	0.19
16	2.81	1.83	4.98	1.93	0.10
17	3.30	0.76	4.16	1.87	0.43
18	3.06	1.25	4.78	1.80	0.23
19	3.23	1.19	4.19	0.78	0.34
20	3.04	1.22	4.50	1.58	0.30
Mean	3.08	1.29	3.02	1.33	0.28

Table S2. Fit parameter values for Race model (Exp. 1).

bioRxiv preprint doi: https://doi.org/10.1101/2023.02.13.528254; this version posted June 5, 2023. The copyright holder for this preprint (which was not certified by peer review) is the author/funder, who has granted bioRxiv a license to display the preprint in perpetuity. It is made available under aCC-BY 4.0 International license.

Subj	κ	u	a	d	μ_{nd}
1	6.37	0.97	4.46	1.16	0.34
2	5.21	1.11	3.73	1.37	0.36
3	7.78	0.59	4.99	3.40	0.39
4	5.97	1.00	-0.87	-0.77	0.38
5	6.24	2.31	1.59	-0.21	0.34
6	4.20	1.59	1.52	1.72	0.34
7	5.87	2.13	0.77	-0.06	0.39
8	4.87	2.24	0.42	-0.43	0.20
9	5.20	1.30	-0.14	1.05	0.41
10	6.40	1.23	4.13	1.37	0.34
11	5.14	1.25	4.36	1.75	0.14
12	4.70	2.03	1.12	0.88	0.33
13	6.25	1.14	4.25	1.46	0.47
14	4.88	2.30	0.76	0.02	0.23
15	5.65	0.87	4.88	1.54	0.26
16	4.38	1.47	4.64	1.70	0.24
17	5.21	0.87	0.91	1.34	0.42
18	4.89	0.97	5.00	1.61	0.33
19	5.05	1.05	3.17	0.67	0.40
20	5.07	1.41	0.81	1.53	0.33
Mean	5.47	1.39	2.52	1.06	0.33

Table 1. Fit parameter values for Difference model (Exp. 1).

Subj	κ	u	a	d	μ_{nd}	B_{mini}
1	7.10	0.87	2.52	1.43	0.32	1.20
2	4.89	0.98	4.72	1.29	0.28	1.34
3	7.16	0.63	-0.85	-1.44	0.37	0.73
4	6.00	0.99	4.06	1.47	0.37	0.18
5	5.99	1.84	2.33	0.16	0.34	0.66
6	3.73	1.76	1.36	1.40	0.30	1.40
7	6.11	1.74	1.18	0.42	0.39	0.95
8	4.73	1.15	0.58	1.80	0.19	1.12
9	3.97	0.69	4.90	3.72	0.42	0.32
10	6.16	1.26	3.45	1.34	0.32	1.17
11	5.11	1.17	4.54	1.68	0.12	1.41
12	4.99	2.05	1.26	0.84	0.35	1.25
13	5.79	1.17	2.66	1.47	0.43	1.22
14	5.21	1.56	1.03	0.95	0.25	0.97
15	5.70	0.86	4.56	1.77	0.21	1.03
16	4.49	1.49	3.69	1.64	0.13	1.82
17	3.50	2.72	2.70	-0.79	0.41	0.78
18	4.92	1.34	5.00	1.43	0.36	0.16
19	5.11	3.16	1.92	-0.17	0.33	0.75
20	5.34	0.90	4.83	1.61	0.35	1.16
Mean	5.30	1.42	2.82	1.10	0.31	0.98

Table S3. Fit parameter values for Two-step model (Exp. 1).

bioRxiv preprint doi: https://doi.org/10.1101/2023.02.13.528254; this version posted June 5, 2023. The copyright holder for this preprint (which was not certified by peer review) is the author/funder, who has granted bioRxiv a license to display the preprint in perpetuity. It is made available under aCC-BY 4.0 International license.

Subj	κ	u	a	d	μ_{nd}
1	15.41	0.65	5.00	1.07	0.46
2	14.00	0.84	4.57	1.22	0.41
3	15.18	0.52	0.85	2.30	0.42
4	14.49	0.56	1.57	3.09	0.41
5	15.16	0.86	3.17	0.43	0.39
6	11.71	2.74	0.92	0.18	0.28
7	14.14	1.67	0.98	-0.07	0.44
8	12.98	1.13	0.96	0.99	0.24
9	14.27	0.67	0.66	1.62	0.44
10	14.71	0.96	2.70	1.20	0.38
11	13.70	0.92	5.00	1.59	0.24
12	12.98	1.44	1.32	0.78	0.45
13	14.84	0.84	4.87	1.29	0.53
14	13.35	2.03	0.87	-0.32	0.30
15	15.48	0.70	2.64	1.73	0.30
16	12.61	1.10	4.23	1.56	0.36
17	14.51	1.41	0.74	-0.57	0.45
18	13.14	0.74	2.99	1.54	0.38
19	13.17	1.60	2.44	0.13	0.38
20	14.15	0.85	2.45	1.32	0.43
Mean	14.00	1.11	2.45	1.05	0.38

Table S4. Fit parameter values for Absolute momentary evidence model (Exp. 1).

Subj	κ	u	a	d	μ_{nd}
1	7.05	1.09	1.34	0.14	0.37
2	6.07	2.06	0.50	-0.09	0.29
3	7.17	2.89	2.45	0.18	0.44
Mean	6.77	2.01	1.43	0.07	0.37

Table S5. Fit parameter values for Difference model in reaction time task (Exp. 2).

Subj	κ	u	a	d
1	10.41	0.17	0.15	9.27
2	9.27	14.97	0.49	3.32
3	8.63	0.36	0.94	4.25
Mean	9.44	5.17	0.53	5.61

Table S6. Fit parameter values for Difference model in controlled duration task (Exp. 2). The high u parameter for subject 2 implies that the decision process was not bounded.

Subj	κ	u	a	d	μ_{nd}
1	5.17	2.17	4.79	0.35	0.38
2	3.67	2.84	1.01	0.56	0.35
3	4.83	5	3.33	0.27	0.53
Mean	4.56	3.34	3.04	0.39	0.42

Table S7. Fit parameter values for Confidence model in reaction time task (Exp. 2).
