# Supplementary material for: Judging the difficulty of perceptual decisions
Source: bioRxiv. 2023 Feb 13:2023.02.13.528254. Preprint. [Version 1] doi: 10.1101/2023.02.13.528254 (PMC9949003; doi:10.1101/2023.02.13.528254)
Supplement: Supplementary file 1 [file NIHPP2023.02.13.528254v1-supplement-1.pdf]

bioRxiv preprint doi: https://doi.org/10.1101/2023.02.13.528254; this version posted February 14, 2023. The copyright holder for this preprint (which was not certified by peer review) is the author/funder, who has granted bioRxiv a license to display the preprint in perpetuity. It is made available under aCC-BY 4.0 International license.

Supporting Information

.

Subj	κ	u	a	d	C_0	μ_{nd}
1	5.89	2.88	0.17	-2.41	-0.02	0.39
2	4.50	2.73	0.22	-0.15	0.10	0.41
3	4.67	1.57	1.23	1.39	-0.02	0.48
4	4.89	1.03	6.00	3.45	0.02	0.49
5	4.01	0.91	6.00	2.92	-0.05	0.51
6	1.34	2.38	0.74	2.90	0.05	0.40
7	4.92	3.80	0.28	-3.00	-0.04	0.43
8	5.36	2.23	0.39	0.68	0.07	0.41
9	2.97	1.82	-0.16	1.76	0.03	0.51
10	5.20	1.25	0.88	4.21	0.01	0.39
11	6.25	1.26	1.06	3.70	0.05	0.37
12	4.55	4.53	0.94	-0.88	-0.03	0.42
13	5.82	4.92	0.36	-1.65	0.02	0.53
14	4.72	0.82	6.00	3.44	0.03	0.55
15	4.27	1.21	0.96	2.72	-0.02	0.42
16	4.53	1.19	1.39	4.18	-0.02	0.51
17	4.23	4.82	0.50	-3.00	0.06	0.50
18	5.73	3.29	0.36	-3.00	-0.08	0.42
19	5.21	5.00	0.65	-1.35	0.00	0.38
20	4.87	3.14	-0.24	5.00	0.10	0.53
Mean	4.70	2.54	1.39	1.05	0.01	0.45

Table S1. Fit parameter values for drift diffusion model of color judgments (Exp. 1a).

Subj	κ	u	a	d	μ_{nd}
1	3.98	1.22	5.00	1.31	0.25
2	3.65	1.50	4.64	1.54	0.19
3	2.97	0.97	-1.02	-0.49	0.40
4	3.88	1.54	-0.81	-0.13	0.36
5	3.82	1.18	2.45	0.73	0.34
6	1.94	1.46	3.34	1.90	0.38
7	3.20	1.63	1.08	1.11	0.32
8	3.53	1.18	2.47	4.00	0.19
9	2.15	0.72	-1.50	-1.75	0.42
10	2.64	1.29	4.78	1.45	0.27
11	2.40	1.36	4.98	1.94	0.11
12	2.76	1.63	1.56	1.67	0.32
13	2.50	1.39	4.85	1.55	0.21
14	2.92	1.36	1.15	1.93	0.24
15	3.76	1.12	4.79	1.87	0.19
16	2.81	1.83	4.98	1.93	0.10
17	3.30	0.76	4.16	1.87	0.43
18	3.06	1.25	4.78	1.80	0.23
19	3.23	1.19	4.19	0.78	0.34
20	3.04	1.22	4.50	1.58	0.30
Mean	3.08	1.29	3.02	1.33	0.28

Table S2. Fit parameter values for Race model (Exp. 1).

bioRxiv preprint doi: https://doi.org/10.1101/2023.02.13.528254; this version posted February 14, 2023. The copyright holder for this preprint (which was not certified by peer review) is the author/funder, who has granted bioRxiv a license to display the preprint in perpetuity. It is made available under aCC-BY 4.0 International license.

Subj	κ	u	a	d	μ_{nd}	B_{mini}
1	7.10	0.87	2.52	1.43	0.32	1.20
2	4.89	0.98	4.72	1.29	0.28	1.34
3	7.16	0.63	-0.85	-1.44	0.37	0.73
4	6.00	0.99	4.06	1.47	0.37	0.18
5	5.99	1.84	2.33	0.16	0.34	0.66
6	3.73	1.76	1.36	1.40	0.30	1.40
7	6.11	1.74	1.18	0.42	0.39	0.95
8	4.73	1.15	0.58	1.80	0.19	1.12
9	3.97	0.69	4.90	3.72	0.42	0.32
10	6.16	1.26	3.45	1.34	0.32	1.17
11	5.11	1.17	4.54	1.68	0.12	1.41
12	4.99	2.05	1.26	0.84	0.35	1.25
13	5.79	1.17	2.66	1.47	0.43	1.22
14	5.21	1.56	1.03	0.95	0.25	0.97
15	5.70	0.86	4.56	1.77	0.21	1.03
16	4.49	1.49	3.69	1.64	0.13	1.82
17	3.50	2.72	2.70	-0.79	0.41	0.78
18	4.92	1.34	5.00	1.43	0.36	0.16
19	5.11	3.16	1.92	-0.17	0.33	0.75
20	5.34	0.90	4.83	1.61	0.35	1.16
Mean	5.30	1.42	2.82	1.10	0.31	0.98

Table S3. Fit parameter values for Two-step model (Exp. 1).

bioRxiv preprint doi: https://doi.org/10.1101/2023.02.13.528254; this version posted February 14, 2023. The copyright holder for this preprint (which was not certified by peer review) is the author/funder, who has granted bioRxiv a license to display the preprint in perpetuity. It is made available under aCC-BY 4.0 International license.

Subj	κ	u	a	d	μ_{nd}
1	15.41	0.65	5.00	1.07	0.46
2	14.00	0.84	4.57	1.22	0.41
3	15.18	0.52	0.85	2.30	0.42
4	14.49	0.56	1.57	3.09	0.41
5	15.16	0.86	3.17	0.43	0.39
6	11.71	2.74	0.92	0.18	0.28
7	14.14	1.67	0.98	-0.07	0.44
8	12.98	1.13	0.96	0.99	0.24
9	14.27	0.67	0.66	1.62	0.44
10	14.71	0.96	2.70	1.20	0.38
11	13.70	0.92	5.00	1.59	0.24
12	12.98	1.44	1.32	0.78	0.45
13	14.84	0.84	4.87	1.29	0.53
14	13.35	2.03	0.87	-0.32	0.30
15	15.48	0.70	2.64	1.73	0.30
16	12.61	1.10	4.23	1.56	0.36
17	14.51	1.41	0.74	-0.57	0.45
18	13.14	0.74	2.99	1.54	0.38
19	13.17	1.60	2.44	0.13	0.38
20	14.15	0.85	2.45	1.32	0.43
Mean	14.00	1.11	2.45	1.05	0.38

Table S4. Fit parameter values for Absolute momentary evidence model (Exp. 1).

Subj	κ	u	a	d	μ_{nd}
1	7.05	1.09	1.34	0.14	0.37
2	6.07	2.06	0.50	-0.09	0.29
3	7.17	2.89	2.45	0.18	0.44
Mean	6.77	2.01	1.43	0.07	0.37

Table S5. Fit parameter values for Difference model in reaction time task (Exp. 2).

Subj	κ	u	a	d
1	10.41	0.17	0.15	9.27
2	9.27	14.97	0.49	3.32
3	8.63	0.36	0.94	4.25
Mean	9.44	5.17	0.53	5.61

Table S6. Fit parameter values for Difference model in controlled duration task (Exp. 2). The high u parameter for subject 2 implies that the decision process was not bounded.

Subj	κ	u	a	d	μ_{nd}
1	5.17	2.17	4.79	0.35	0.38
2	3.67	2.84	1.01	0.56	0.35
3	4.83	5	3.33	0.27	0.53
Mean	4.56	3.34	3.04	0.39	0.42

Table S7. Fit parameter values for Confidence model in reaction time task (Exp. 2).

bioRxiv preprint doi: https://doi.org/10.1101/2023.02.13.528254; this version posted February 14, 2023. The copyright holder for this preprint (which was not certified by peer review) is the author/funder, who has granted bioRxiv a license to display the preprint in perpetuity. It is made available under aCC-BY 4.0 International license.

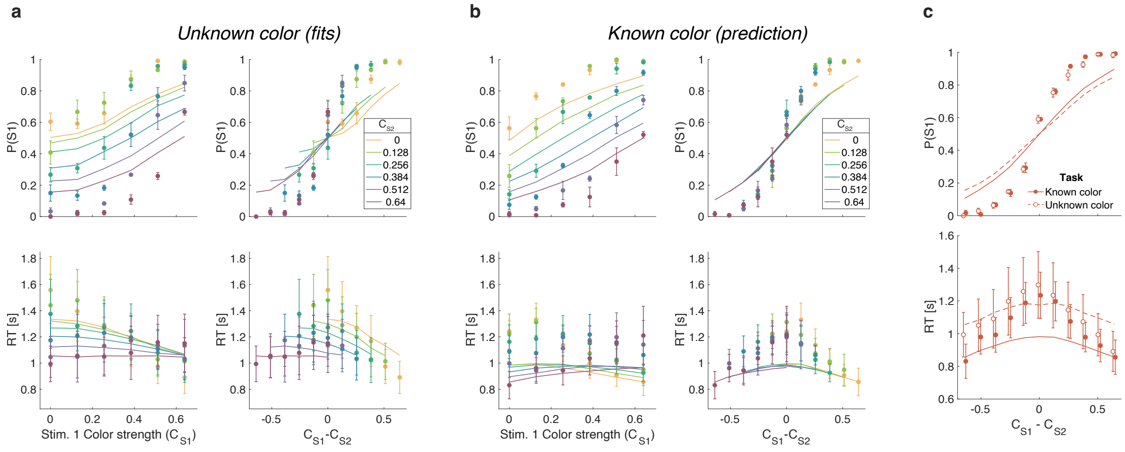

Figure 6—figure supplement 1. As main figure but with lines showing the fit of the Confidence model to the unknown color condition and predictions for the known color condition.
